# Circular

**Published:** 1868-03-01

**Authors:** E. W. Moore

**Affiliations:** Decatur, Ill.


### *Circular.*

Attention is directed to the following Circular, which explains itself:

DECATUR, ILL., Jan. 23d, 1868.

Having been appointed Chairman of the Committee on Obstetrics, by the Illinois State Medical Society, at its last annual session, I would respectfully and earnestly solicit your aid in furnishing material for Report.

A timely answer to any or all the following questions will be gratefully received and duly credited.

I desire to know:

I. The number of births that have occurred within your knowledge during the past year.

II. Number of males and females, and weight of each.

III. Number of abortions and premature labors, with causes which induced them.

IV. Number of still-born children, male and female, with causes. State time of child's death, whether before or during labor—if before, how long.

V. Number of children, male and female, born alive, who have died within twenty-four hours after birth.

VI. Number of cases prolonged *gestation*, with number of days beyond the ordinary period.

VII. Number of monstrosities.

VIII. Number of multiple births, with sex in each case.

IX. Number of cases of instrumental labor, with kind of instrument used.

X. Number of complicated labors, with nature of complication and results.

XI. Number of cases of hæmorrhage, before, during, or after labor, whether concealed or otherwise, with results.

XII. Number of cases of *adherent placenta*, with results.

XIII. Number of cases of *puerperal convulsions* occurring before, during, or after labor, with outline of treatment and results.

XIV. Number of cases of *puerperal mania*, with causes, treatment and results.

XV. Number of cases *puerperal fever*, with outline of treatment and results.

XVI. Number of cases *cellulitis*, with cause, treatment and results.

XVII. Number of cases of rupture of *womb*, *vagina* or *perineum*, with treatment and results.

XVIII. Number of new remedies applicable to puerperal women.

XIX. Number of new obstetrical instruments, or new improvements of old ones.

XX. Number cases of *atresia vaginæ*, with causes, treatment and results.

XXI. Number cases of inversion of uterus, with causes, treatment and results.

Any thing of unusual interest connected with Obstetrics, not included in or suggested by the foregoing, will be most gratefully received.

A prompt answer is very desirable. Every thing that will appear in the Report must reach me by the 1st, or at farthest, by the 15th of March.

E. W. MOORE, M.D.

